# The Potential Role of Cigarette Smoke, Elastic Fibers, and Secondary Lung Injury in the Transition of Pulmonary Emphysema to Combined Pulmonary Fibrosis and Emphysema

**DOI:** 10.3390/ijms252111793

**Published:** 2024-11-02

**Authors:** Jerome Cantor

**Affiliations:** School of Pharmacy and Allied Health Sciences, St John’s University, Queens, NY 11439, USA; cantorj@stjohns.edu

**Keywords:** pulmonary fibrosis, pulmonary emphysema, cigarette smoke, lipopolysaccharide, elastin, desmosine

## Abstract

Combined pulmonary fibrosis and emphysema (CPFE) is a distinct syndrome associated with heavy smoking. The fibrotic component of the disease is generally believed to be superimposed on previously existing pulmonary emphysema, but the mechanisms responsible for these changes remain poorly understood. To better understand the pathogenesis of CPFE, we performed a series of experiments that focused on the relationships between lung elastic fibers, cigarette smoke, and secondary lung injury. The results indicate that even brief smoke exposure predisposes the lung to additional forms of lung injury that may cause alveolar wall fibrosis. The proinflammatory activity of smoke-induced structural alterations in elastic fibers may contribute to this process by enhancing secondary lung inflammation, including acute exacerbations of chronic obstructive pulmonary disease. Furthermore, the levels of the unique elastin crosslinks, desmosine and isodesmosine, in blood, urine, and sputum may serve as biomarkers for the transition from pulmonary emphysema to interstitial fibrosis. While the long-term effects of these inflammatory reactions were not examined, the current studies provide insight into the potential relationships between elastic fiber injury, cigarette smoke, and secondary lung injury. Determining the mechanisms involved in combined pulmonary emphysema and fibrosis and developing a sensitive biomarker for this type of lung injury may permit timely therapeutic intervention that could mitigate the high risk of respiratory failure associated with this condition.

## 1. Introduction

While pulmonary emphysema is commonly associated with distended airspaces and alveolar wall rupture, a number of studies indicate that these morphological features may be accompanied by areas resembling pulmonary fibrosis. The difficulty of classifying lung disease with airspace enlargement that may be accompanied by alveolar wall thickening was at least partially resolved by creating a new category known as combined pulmonary fibrosis and emphysema (CPFE) [1,2,3,4]. In contrast to the cystic airspace enlargement seen in pulmonary fibrosis, CPFE shows morphological features associated with emphysema, including the distention and rupture of alveolar walls. While another entity, respiratory bronchiolitis with fibrosis, may have comparable morphological findings, the fibrosis is usually localized around bronchioles rather than diffusely spread throughout the lung, and may involve different mechanisms [5].

The pathogenesis of CPFE is not well understood but may relate to the effects of cigarette smoke, which can induce both airspace enlargement and interstitial fibrosis. The current paper provides support for the hypothesis that smoke exposure combined with structurally altered elastic fibers and secondary lung injury produces an inflammatory process that superimposes fibrotic lesions on previously existing distended and ruptured alveolar walls, resulting in a complex pattern of pulmonary emphysema and interstitial fibrosis.

The proximity of alveolar wall fibrosis to airspace enlargement in CPFE suggests that both entities may have a common origin [2]. The fragmentation of elastic fibers due to elastases and mechanical strain may attract inflammatory cells that release various mediators responsible for fibrogenesis [6]. Consequently, the co-existence of pulmonary emphysema and interstitial fibrosis may represent a sequence of related events rather than distinct disease processes.

This hypothesis is supported by a study involving bleomycin (BLM)-treated α1-proteinase inhibitor-deficient mice, which demonstrated that emphysema-related airspace enlargement precedes the development of interstitial fibrosis [7]. Both disease processes involved increased neutrophil elastase activity and could be significantly attenuated by using a neutrophil elastase inhibitor. These findings suggest that neutrophil elastase may be a mechanistic component in the pathogenesis of both pulmonary emphysema and pulmonary fibrosis, responsible for the degradation and subsequent remodeling of the extracellular matrix. The dual role of elastase may involve the synthesis of both cytokines and their associated receptors that regulate inflammatory cell recruitment, epithelial–mesenchymal transition, and other cellular events [8].

The co-existence of pulmonary emphysema and interstitial fibrosis may also be related to superimposed lung injury. Studies performed in our laboratory investigated the effects of secondary lipopolysaccharide (LPS)-induced injury in animal models involving either short-term cigarette smoke exposure or elastase-induced emphysema. The results indicate that the inflammatory events associated with these models synergistically interact with superimposed injury, resulting in markedly increased lung disease. Based on these findings, we propose that secondary injury, including respiratory infections associated with acute exacerbations of chronic obstructive pulmonary disease (AECOPD), may play an important role in the pathogenesis of CPFE. This finding provides further support for the concept that CPFE results from initial alveolar wall injury and rupture that evolves into interstitial fibrosis by a sequence of events involving secondary injury due to cigarette smoke or other toxic agents.

## 2. The Role of Elastic Fibers in Pulmonary Emphysema

**Figure 1:**
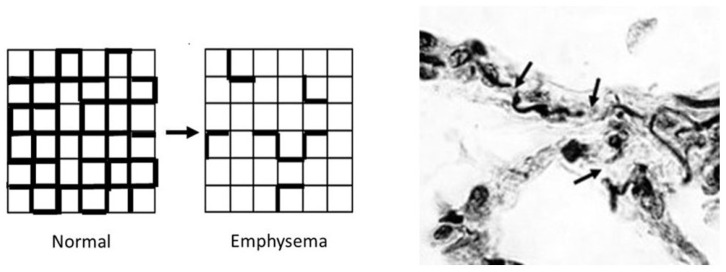
(**Left**) The development of pulmonary emphysema involves a loss of structurally intact elastic fibers (bold lines). The mechanism responsible for this change involves enzymatic and oxidative degradation of the fibers, which increases the mechanical strain on alveolar walls, resulting in their distention and rupture. (**Right**) Photomicrograph of postmortem lung from a smoker with moderate pulmonary emphysema showing fragmentation of elastic fibers (arrows). Reprinted with permission [21]. Original magnification: 1000×.

The development of airspace enlargement in pulmonary emphysema involves multiple mechanisms acting on various lung components. However, a major component of the disease is damage to lung elastic fibers [9]. Inflammatory cells recruited by cigarette smoke and other toxins release elastases and oxidants that degrade these fibers, causing disruption of the mechanical forces responsible for the movement of air through the lung (Figure 1) [8]. The breakdown of these fibers leads to the distention and rupture of alveolar walls, which reduces lung surface area and impairs gas exchange.

While increased elastase activity plays a central role in this process, other mechanisms may be more directly responsible for the morphological changes associated with pulmonary emphysema. Alterations in the transmission of mechanical forces may be needed to convert proteolytic injury into airspace enlargement [10,11,12,13,14]. This concept is supported by in silico studies showing that focal variations in alveolar wall elasticity progress to widespread architectural changes resembling those seen in pulmonary emphysema [14].

The mechanical characteristics of elastic fibers are based on their capacity to store and release energy by changing their configuration. During inspiration, distention of these fibers results in a more ordered arrangement that decreases entropy, while expiration is associated with the return to a more disordered state, providing the mechanical recoil required to expel air from the lungs. These changes in entropy are dependent on the properties of the core elastin protein, which contains hydrophobic regions that interact with adjacent water molecules [13].

The unique crosslinks, desmosine and isodesmosine (DID), are responsible for the structural integrity of elastin. They are synthesized by the condensation of lysyl residues in neighboring peptide chains [11]. The critical role of DID and other crosslinks was demonstrated by the use of the crosslinking inhibitor beta-aminopropionitrile to modify cadmium chloride-induced pulmonary fibrosis [15]. Treatment with this agent resulted in the development of pulmonary emphysema rather than interstitial fibrosis.

As a result of the low turnover of elastic fibers in the absence of lung injury, DID may serve as a biomarker for pulmonary emphysema. Increases in DID are seen in the blood, urine, and sputum of patients with pulmonary emphysema, and plasma levels correlate with a decrease in lung mass, as measured by high-resolution CT imaging [16,17,18,19].

The lung content of peptide-free DID was measured in hamsters treated with cigarette smoke, followed by intraperitoneally administered LPS to induce pulmonary emphysema [20]. A significant correlation between peptide-free lung DID and increasing alveolar diameter was observed, indicating that this parameter may serve as a biomarker for airspace enlargement. Subsequent measurements of DID in normal and emphysematous postmortem human lungs showed that the density of these crosslinks in lung tissue sections underwent a marked increase when the alveolar diameter exceeded 300 µm, and they leveled off at 400 µm [21]. These findings indicate that the initial stages of airspace distention involve a balance between injury and repair of elastic fibers, where increased crosslink density is associated with enhanced deposition of elastin.

## 3. The Effects of Long-Term Smoke Exposure on Pulmonary Elastic Fibers

**Figure 2:**
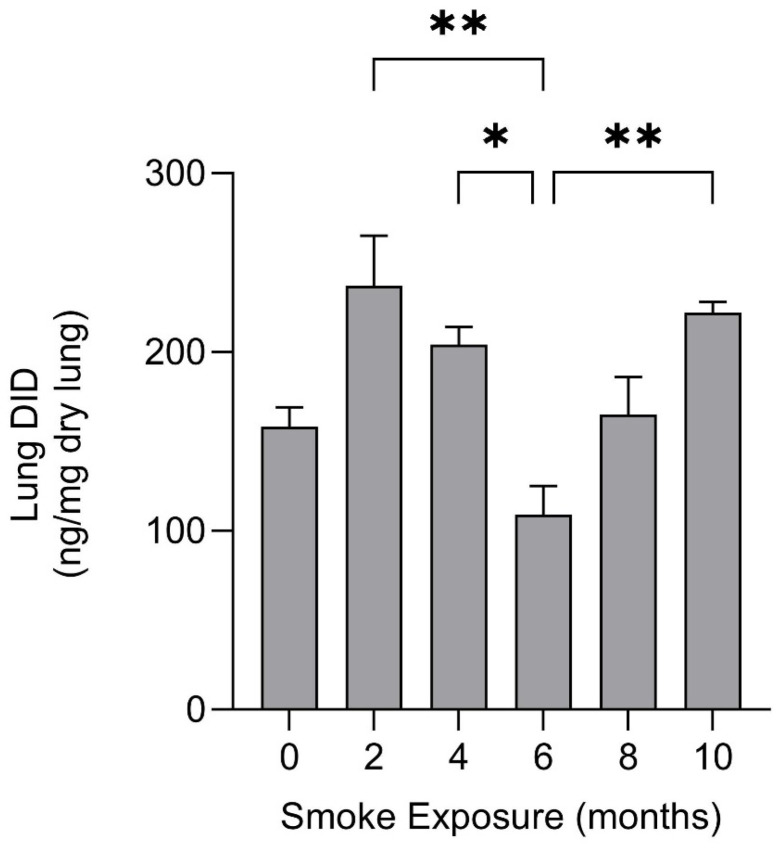
Total lung DID was increased at two months, then markedly decreased over the next four months before undergoing a secondary increase over the remaining course of the study [22]. N ≥ 3 for each group. T-bars indicate the standard error of the mean (SEM). The number of asterisks above the bars correlates with the level of statistical significance (*p* < 0.05, *p* < 0.01).

**Figure 3:**
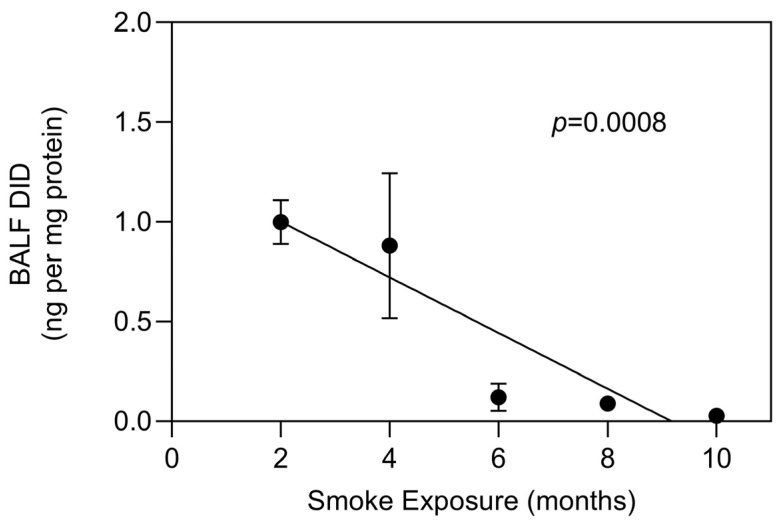
The decrease in total lung DID content shown in Figure 2 is reflected by relatively high levels of BALF DID, whereas the subsequent increase in lung DID is associated with a marked decline in BALF DID 22]. N ≥ 3 for each group. T-bars indicate SEM.

Our laboratory studied the effect of a ten-month exposure to cigarette smoke on mouse lung elastic fibers [22]. Total lung DID was initially increased at two months, then markedly decreased over the next four months before undergoing a secondary increase over the remaining course of the study, suggesting changes in the balance between elastic fiber injury and repair (Figure 2). This concept is further supported by markedly decreased BALF DID levels at 6 months, consistent with reduced elastin breakdown as the repair of elastic fibers progresses (Figure 3).

After several months, there was a leveling off of airspace enlargement in the smoke-exposed mice, consistent with an adaptive response to continued lung injury. This may be due to the enhanced synthesis of endogenous antioxidants that limit the adverse effects of tobacco smoke and other oxidants [23,24]. Changes in the interstitial extracellular matrix caused by injury and repair could decrease alveolar wall rupture due to mechanical stress. Regarding this possibility, an increase in lung collagen content has been reported after prolonged exposure to cigarette smoke, suggesting a similar transition to a proliferative process that maintains alveolar wall structural integrity [25].

## 4. The Proinflammatory Activity of Structurally Modified Elastic Fibers

The relationship between lung inflammation and elastic fiber injury was studied in a hamster model involving the intratracheal (rather than intraperitoneal) instillation of LPS to augment airspace enlargement previously induced by intratracheal elastase [6]. In contrast to studies using multiple weekly treatments with elastase before LPS, this model employed a single low dose of the enzyme and reduced the time period between the instillation of the two agents to a single week [26,27]. This increased the relative effect of LPS compared to elastase and permitted the identification of synergistic interactions between the two agents.

This model was used to determine whether pretreatment with elastase changed the structure of elastic fibers, increasing their susceptibility to secondary injury due to LPS. The effect of intratracheally instilled elastin peptides on the LPS-induced recruitment of leukocytes was also examined to ascertain the proinflammatory role of damaged elastic fibers.

**Figure 4:**
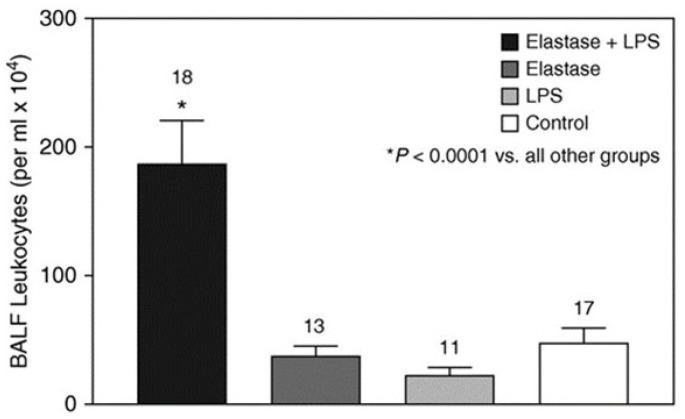
The intratracheal administration of elastase before the instillation of LPS results in a significantly higher level of BALF leukocytes than the other treatment groups [6]. The numbers above the bars indicate N. T-bars denote SEM. Reprinted with permission.

**Figure 5:**
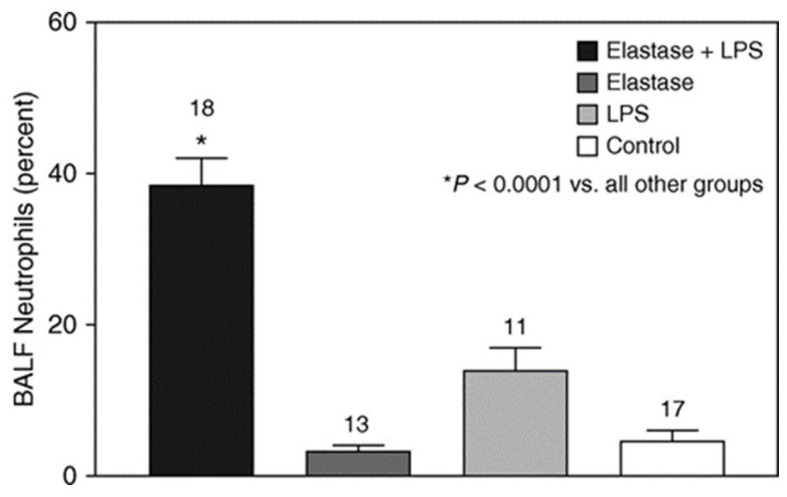
The intratracheal administration of elastase prior to the instillation of LPS results in a significantly higher level of BALF neutrophils compared to the other treatment groups [6]. The numbers above the bars indicate N. T-bars denote SEM. Reprinted with permission.

Total BALF leukocytes and percent neutrophils were measured two days after the instillation of LPS. Hamsters treated with elastase and LPS had a significantly increased number of BALF cells compared to those given elastase/saline, saline/LPS, or controls (Figure 4). The percentage of BALF neutrophils was also significantly higher in animals receiving elastase and LPS compared to those given elastase or LPS alone (Figure 5).

**Figure 6:**
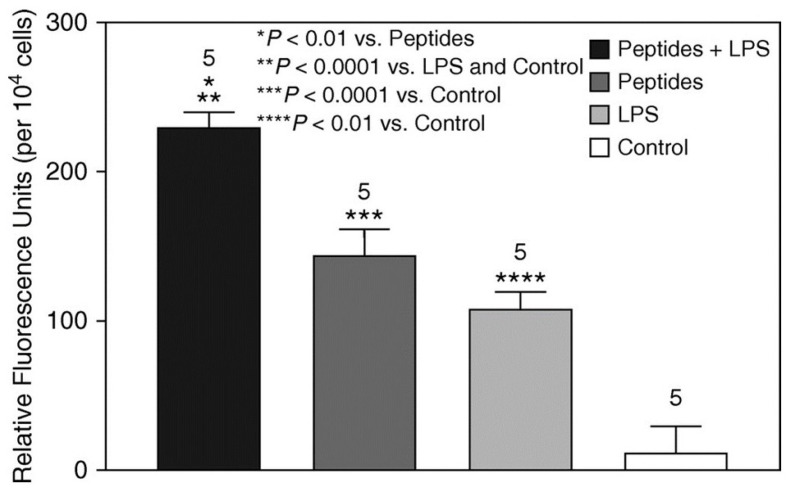
The combination of elastin peptides and LPS had a significantly greater chemoattractive effect on BALF macrophages compared to the other treatment groups [6]. Chemotattraction was measured by labeling cells with a fluorescent dye. The numbers above the bars indicate N. T-bars denote SEM. Reprinted with permission.

The chemoattractive activity of elastin peptides and LPS was measured using BALF cells from untreated animals composed almost exclusively of macrophages. Exposure to either elastin peptides or LPS alone significantly increased cell mobility compared with controls (Figure 6). However, combining peptides with LPS had a much greater chemoattractive effect, possibly due to increased macrophage activation.

These findings support the concept that elastase has multiple roles in the pathogenesis of CPFE. The degradation of elastic fibers causes the release of elastin peptides, which attract inflammatory cells, bind to elastin receptor complexes, and induce various cellular activities, including elastogenesis, protease activation, and apoptosis [7]. While these events may not be directly related to the development of interstitial fibrosis, they nevertheless produce changes in the alveolar wall extracellular matrix (ECM) that can induce fibrogenesis [8].

**Figure 7:**
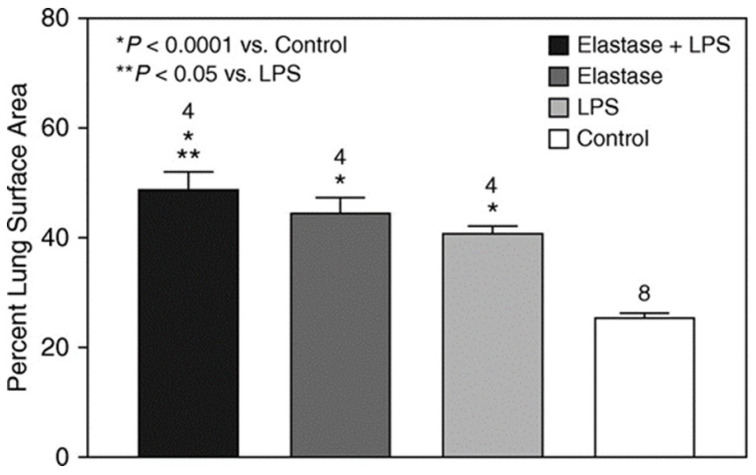
The intratracheal administration of elastase prior to the instillation of LPS results in a significantly greater elastic fiber surface area compared to treatment with LPS alone, consistent with increased proinflammatory structural alterations in these fibers [6]. The numbers above the bars indicate N. T-bars denote SEM. Reprinted with permission.

Interstitial elastic fiber surface area was determined one week after LPS. Despite increased lung elastin degradation (as determined by the level BALF DID), animals treated with elastase and LPS had significantly greater elastic fiber surface area than the LPS-only or control groups (Figure 7). The increase in elastic fiber surface area might result from either a reduced number of DID crosslinks following elastase treatment or impaired crosslink formation during the repair process, both of which may cause the splaying of the fibers. This hypothesis is supported by a study of patients with severe COPD that showed significantly increased elastic fiber surface area without an accompanying increase in desmosine content [28].

While the concentration of elastase used in this study was lower than that used by other investigators to induce emphysema in hamsters, it caused sufficient elastic fiber damage to produce a synergistic effect with LPS [6,29]. This result indicates that structural changes in the fibers may enhance secondary inflammation, leading to further alveolar wall injury that results in a proliferation of elastic fibers and other connective tissue components [6]. This mechanism could be further investigated in the current model and may explain the relationship between AECOPD and the loss of lung function [4]. It is anticipated that additional treatment with LPS, which mimics the effect of acute exacerbations, may increase the extent of interstitial fibrosis.

The effect of fragmented elastic fibers on the recruitment of inflammatory cells is supported by studies from other laboratories showing that elastin breakdown products act as leukocyte chemoattractants in vitro, and the intratracheal instillation of specific elastin peptides induces an inflammatory response that involves the remodeling of the interstitial ECM [6,30].

## 5. Cigarette Smoke Predisposes the Lungs to Secondary Injury

### 5.1. The Effect of Cigarette Smoke on Amiodarone-Induced Lung Injury

Although the long-term inhalation of cigarette smoke is associated with COPD, the more immediate effects are not well understood [31]. Brief exposure to smoke does not cause significant pulmonary injury and may adversely affect the lung only if there is underlying disease present [26,32]. This may be particularly true of second-hand smoke, which could require the presence of secondary lung injury to induce an inflammatory response.

To test these hypotheses, hamsters were briefly exposed to cigarette smoke (2 h per day × 5 days) either before or after the intratracheal instillation of amiodarone, an antiarrhythmic agent that induces lung injury [33]. In both cases, the short-term inhalation of smoke enhanced various parameters of pulmonary inflammation, including histologically graded injury, BALF neutrophil content, TNFR1-positive BALF macrophages, and alveolar cell apoptosis. Furthermore, the effects of cigarette smoke and amiodarone were consistent with a synergistic interaction between these agents.

**Figure 8:**
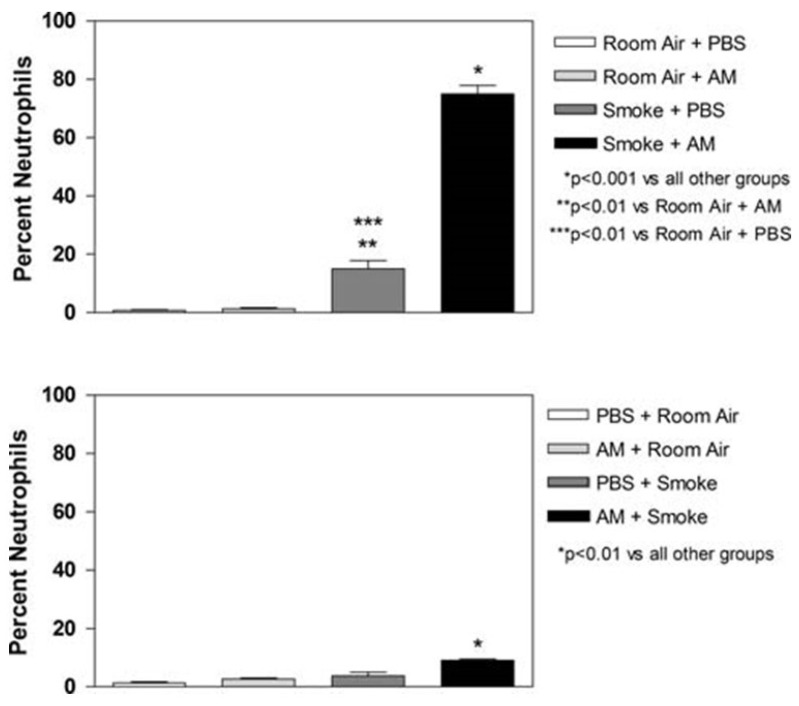
Exposure to cigarette smoke prior to intratracheal instillation of Amiodarone (upper graph) results in a significantly greater influx of neutrophils into the lung compared to post-treatment with smoke (lower graph) [33]. N = 3 for each group. T-bars indicate SEM. Reprinted with permission.

The results show that both pre-exposure and post-exposure to cigarette smoke potentiate amiodarone-induced lung injury. However, pretreatment with smoke had a greater effect, especially with regard to the influx of neutrophils into the lungs (Figure 8). Compared to post-treatment, there was a 733% increase in the percentage of BALF neutrophils, a 112% increase in total BALF leukocytes, a 144% increase in TNFR1-labeled macrophages, and a 38% increase in alveolar septal cell apoptosis.

This asymmetry may depend on the differential effects of cigarette smoke and amiodarone. Cigarettes contain numerous toxins that may cause much more extensive inflammatory changes than those associated with amiodarone [34]. Thus, exposure to cigarette smoke prior to LPS is more likely to produce synergistic interactions with a second injurious agent. In contrast, pretreatment with LPS may activate a more limited set of inflammatory processes, reducing the degree of synergy with cigarette smoke.

The most striking difference between the pre- and post-smoked groups involved the influx of neutrophils in the lung. The marked increase in BALF neutrophils associated with pre-exposure to smoke is indicative of the increased migration of these cells from the pulmonary capillary network to the lung interstitium, which may result from the increased secretion of neutrophil elastase. It was previously shown that a deficiency in this enzyme significantly reduces the smoke-induced movement of neutrophils into the extravascular space, suggesting that elastase facilitates the dissociation of these cells from adhesion molecules that tether them to capillary endothelium [35]. Pre-exposure to smoke may increase the capacity of these cells to respond to chemoattractants generated by the instillation of amiodarone. In contrast, the proinflammatory effects of post-treatment with smoke may be limited by the rapid clearance of amiodarone from the lung.

The synergistic interaction of smoke with amiodarone may also involve tumor necrosis factor (TNF)-alpha, which can induce the release of proinflammatory mediators by macrophages [36]. Both cigarette smoke and amiodarone are associated with elevated levels of TNF-alpha, and the enhanced expression of TNFR1 by these cells could increase their responsiveness to this cytokine [37,38]. Studies have shown that the development of pulmonary inflammatory infiltrates and extracellular matrix injury following either smoke exposure or LPS administration may be largely due to the effects of TNF-alpha. The increased production of this cytokine induces the transcription of genes responsible for the synthesis of matrix metalloproteinases and the recruitment of neutrophils to the lung [39].

The increase in alveolar septal cell apoptosis following treatment with both cigarette smoke and LPS may also be mediated by TNF-alpha and could play an important role in alveolar wall injury. Furthermore, elastase released by inflammatory cells may induce alveolar epithelial apoptosis by binding to the protease-activated receptor-1 [40]. The loss of these cells may be an important mechanism in the pathogenesis of pulmonary emphysema.

In contrast, leukocyte apoptosis may actually decline following treatment with these agents, which may prolong the inflammatory response [41,42]. Consequently, even brief exposure to second-hand smoke may be potentially harmful to individuals with pre-existing lung disease.

### 5.2. The Effect of Smoke Exposure on LPS-Induced Lung Injury

**Figure 9:**
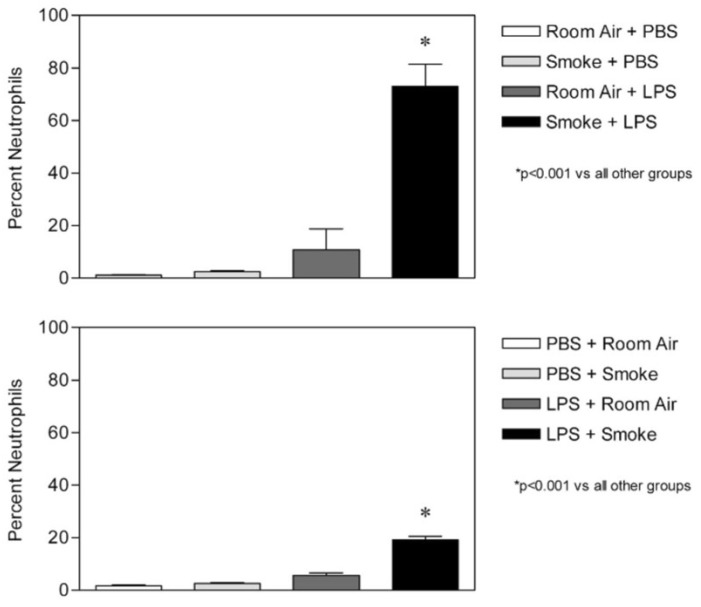
Exposure to cigarette smoke prior to intratracheal instillation of LPS (upper graph) results in a significantly greater influx of neutrophils into the lung compared to post-treatment with smoke (lower graph) [43]. N = 5 for each group. T-bars indicate SEM. Reprinted with permission.

Hamsters were briefly exposed to second-hand cigarette smoke (2 h per day × 3 days) either before or after the intratracheal administration of LPS [43]. Both pre-and post-exposure to smoke potentiated acute pulmonary injury induced by a low dose of LPS. However, pretreatment with smoke had a greater proinflammatory effect than post-treatment, with neutrophils again showing the greatest difference (Figure 9). Compared to post-treatment, there was a 284% increase in the percentage of BALF neutrophils, a 25% increase in TNFRI-labeled macrophages, and a 27% increase in apoptotic cells. The reasons for this disparity in the inflammatory response are unclear but may involve the same processes responsible for the differences seen with Amiodarone. In addition to the mechanisms previously discussed, reactive oxidant species in cigarette smoke may impair elastin crosslinking, causing structural changes in elastic fibers that predispose them to enzymatic breakdown [44].

Morphological changes in the lung also reflected the synergistic interactions between smoke and LPS. Microscopic examination of the lungs of hamsters exposed to smoke (4 h per day × 3 days) prior to intraperitoneal administration of LPS revealed significant inflammatory cell infiltrates resulting in alveolar wall thickening, whereas lungs treated with only smoke or LPS did not show these changes [20].

While our laboratory only investigated the effects of cigarette smoke, other inhaled toxins may be similarly harmful. Further examination of both outdoor and indoor air pollutants is needed to determine their ability to potentiate the effects of secondary lung injury. Synergistic interactions involving even minute amounts of pulmonary toxins may lead to the development of subacute inflammatory processes that result in significant lung injury over time.

## 6. A Potential Biomarker for the Transition from Pulmonary Emphysema to Interstitial Fibrosis

Previously, we investigated elastic fiber injury in experimental models of pulmonary fibrosis and emphysema induced by intratracheal BLM and elastase, respectively [45,46]. While increases in BALF DID were seen in both forms of injury, there was a marked difference between the two with regard to the proportion of peptide-free crosslinks. In elastase-induced pulmonary emphysema, the ratio of free to peptide-bound DID was significantly increased compared to controls, whereas this parameter was decreased from normal in the BLM model of pulmonary fibrosis.

These findings suggest that this ratio may be used to differentiate the extracellular matrix changes in pulmonary emphysema from those associated with interstitial fibrosis. This hypothesis was further tested in a 28-day clinical trial involving the use of aerosolized hyaluronan to prevent elastic fiber injury [47]. Despite the short duration of the study, there was a preferential reduction in the amount of free DID in the urine of COPD patients treated with hyaluronan, indicating the sensitivity of this biomarker as a measure of elastic fiber degradation that may reflect alveolar wall injury.

The proportional decrease in peptide-free DID in pulmonary fibrosis may be associated with the increased deposition of extracellular matrix, which could provide structural support for elastic fibers and prevent their degradation. In particular, an increase in hyaluronan, which binds to elastic fibers, could limit the effects of elastases and other injurious agents.

## 7. Future Directions

CFPE is a lung disease characterized by changes in the structure of the lungs, such as enlarged airspaces and fibrosis. Detecting CFPE at an early stage requires an understanding of the molecular and larger-scale mechanisms that contribute to the development of the disease. Applying a percolation system to model the disease suggests that the most reliable biomarkers of these initial events are those that reflect microarchitecture changes associated with the airspace enlargement that precedes fibrosis. The ratio of free to peptide-bound DID may, therefore, provide an opportunity to monitor this transition. An increase in this parameter would be consistent with the development of interstitial fibrosis.

As shown in the clinical trial of HA, free DID in urine may also be a useful biomarker for the progression of airspace enlargement in CPFE [47]. Despite the potential influence of co-existing diseases involving elastic fiber damage, such as atherosclerosis or osteoarthritis, on the specificity of free DID as a biomarker, they could nevertheless play a critical role in clinical trials. Significant differences in crosslink levels between closely matched experimental and control groups would demonstrate therapeutic efficacy.

Furthermore, measuring free DID in sputum and possibly breath condensate could enhance the specificity for the emphysematous component of CPFE. However, for free DID to be accepted as a biomarker for this condition, it will be necessary to develop an accurate and reproducible analytic method. Currently, there is no standardized protocol for measuring free DID, and the cost of expertise and equipment may pose challenges to the widespread adoption of this biomarker.

**Figure 10:**
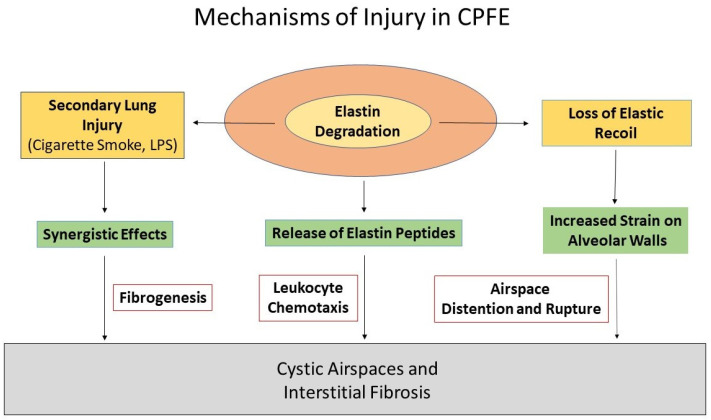
Diagram of the proposed mechanisms involved in the pathogenesis of CPFE.

Regarding the treatment of CPFE, potential agents that target multiple mechanisms involved in the pathogenesis of alveolar wall injury may have increased efficacy. Current treatments that focus on a specific inflammatory process, such as elastase inhibitors, have shown limited effectiveness in treating the disease. This finding may be due to the complex nature of the disease, where various interactions at different levels of scale are primarily responsible for its progression rather than the activity of individual molecules (Figure 10). Consequently, a single drug would be incapable of ameliorating both airspace enlargement and interstitial fibrosis.

This hypothesis is supported by the limited success of previous clinical trials involving various anti-inflammatory agents, such as corticosteroids and elastase inhibitors. Currently, the only effective treatment for pulmonary emphysema is alpha-1 antiproteinase supplementation in a relatively small subset of COPD patients with a genetic deficiency of this protein [48,49,50,51].

Due to the lack of a broadly effective agent for treating the emphysematous component of CPFE, future clinical trials should consider the use of one or more antifibrotic agents in combination with a potential therapy for pulmonary emphysema, such as aerosolized HA. In contrast to pulmonary emphysema, several drugs have shown a moderate degree of success in slowing the progression of interstitial fibrosis. They generally target either epithelial–mesenchymal transition or fibroblast activity. The efficacy of the proposed hybrid drug regimen could be determined by monitoring changes in the ratio of free to peptide-bound DID in urine.

The rationale for including HA is its effect on multiple mechanisms responsible for alveolar wall injury, thereby reducing the likelihood of subsequent fibrogenesis [52]. This concept is supported by a recent study showing that the combination of aerosolized HA with other drugs may have a synergistic effect in the treatment of AECOPD, which occurs more frequently in patients with CPFE and may be a source of secondary lung injury that induces pulmonary fibrosis [53]. This approach could include combining HA with elastase inhibitors or drugs that induce elastic fiber resynthesis.

The importance of crosslinking in maintaining the structural integrity of alveolar walls suggests an alternative form of treatment involving the development of drugs that enhance the activity of the components responsible for this process. In particular, agents that increase the density of DID might alter the balance between injury and repair of elastin. This approach would include the administration of copper to increase the level of lysyl oxidase, the enzyme involved in the synthesis of elastin and collagen crosslinks [54]. Conversely, the therapeutic manipulation of this enzyme with agents that chelate copper might reduce interstitial fibrosis.

While there are no treatment options for restoring normal lung architecture, the proposed implementation of stem cell therapies may eventually offer this possibility. Preliminary studies suggest the potential role of stem cells in immunomodulation and tissue regeneration [55,56,57,58]. The therapeutic advantages of this approach include the release of extracellular vesicles containing lipids, nucleic acids, and proteins that can induce repair of the ECM, potentially reversing the disease process.

Finally, studies demonstrating the effectiveness of endothelin receptor antagonists (ERAs) in ameliorating both experimentally induced and human interstitial fibrosis suggest that treatment with these agents may slow the progression of CPFE [59,60,61,62]. The current treatment of pulmonary hypertension and interstitial fibrosis with ambrisentan and other ERAs indicates the feasibility of using these drugs in clinical trials involving patients with CPFE. However, early detection of the disease may be required to significantly improve survival.

## 8. Conclusions

The combination of brief cigarette smoke exposure with other pulmonary toxins facilitated the identification of potential relationships between structurally altered lung elastic fibers, cigarette smoke, and secondary lung injury that may account for the coexistence of interstitial fibrosis pulmonary emphysema in the lungs of patients with COPD. The effect of elastic fiber fragmentation may be particularly important because of the resulting uneven distribution of pulmonary mechanical forces, which may induce a self-perpetuating process of elastin peptide release, leukocyte chemotaxis, and further alveolar wall injury resulting in both airspace enlargement and interstitial fibrosis.

The association of CPFE with heavy cigarette smoking and AECOPD provides support for the proposed role of synergistic interactions between smoke and other proinflammatory agents in this disease. Nevertheless, further work is needed to validate this concept and determine whether the free to peptide-bound DID ratio reflects the emergence of fibrotic reactions in pulmonary emphysema. The development of a sensitive biomarker for CPFE would permit timely therapeutic intervention that may reduce the high risk of respiratory failure associated with this disorder.
